# Increased Levels of Plasma Soluble Sema4D in Patients with Heart Failure

**DOI:** 10.1371/journal.pone.0064265

**Published:** 2013-05-31

**Authors:** Qiongyu Lu, Ningzheng Dong, Qi Wang, Wenxiu Yi, Yuxin Wang, Shengjie Zhang, Haibo Gu, Xin Zhao, Xiaorong Tang, Boquan Jin, Qingyu Wu, Lawrence F. Brass, Li Zhu

**Affiliations:** 1 Cyrus Tang Hematology Center, Soochow University, Suzhou, Jiangsu, China; 2 Jiangsu Institute of Hematology, Soochow University, Suzhou, Jiangsu, China; 3 Department of Cardiology of The First Affiliated Hospital, Soochow University, Suzhou, Jiangsu, China; 4 Department of Cardiology of The Second Affiliated Hospital, Soochow University, Suzhou, Jiangsu, China; 5 Department of Pathology, Jilin Hospital of Chinese Armed Police Force, Changchun, Jilin, China; 6 Department of Immunology, The Fourth Military Medical University, Xi’an, Shanxi, China; 7 Departments of Medicine and Pharmacology, University of Pennsylvania, Philadelphia, Pennsylvania, United States of America; Thomas Jefferson University, United States of America

## Abstract

Semaphorin 4D (Sema4D/CD100) is a 150-kDa transmembrane glycoprotein expressed by platelets and T-cells. When these cells are activated, Sema4D is cleaved proteolytically, generating a biologically active 120-kDa fragment (soluble Sema4D) capable of targeting receptors on platelets, B-cells, endothelial cells and tumor cells. However, its plasma levels and significance in heart failure (HF) have not been reported. In this study, we established an ELISA and detected soluble Sema4D in human plasma. In healthy controls, plasma Sema4D levels were higher in men than women (5.15±3.30 ng/mL, n = 63, vs. 4.19±2.39 ng/mL, n = 63, *P*<0.05). In HF patients, plasma Sema4D levels were significantly higher than those in healthy controls (8.94±5.89 ng/mL, n = 157 vs. 4.67±2.99 ng/mL, n = 126, *P*<0.0001) with the highest levels being in HF patients with diabetes mellitus (DM) (10.45±5.76 ng/mL, n = 40). We also found that there was a higher percentage of Sema4D^high^ CD3^+^ (*P*<0.01), CD4^+^ (*P*<0.001), and CD8^+^ (P<0.01) T-cells in samples from HF patients, but no changes in Sema4D expression levels in B cells and platelets. Therefore, our investigation shows that plasma Sema4D levels are increased in HF patients, especially in those who also have diabetes. There was an accompanying increase in the Sema4D^high^ population of T-cells, suggesting a potential role of these T-cells in heart failure.

## Introduction

Sema4D (or CD100) is a 150-kDa type I integral membrane glycoprotein and a member of the class IV semaphorin family. It was first reported to be expressed on T cells where it supports B cell development by binding to CD72, a low affinity receptor associated with the protein tyrosine phosphatase SHP-1 [1]. Subsequent studies show that Sema4D participates in axon guidance [2], [3], [4], [5], angiogenesis [6], [7], [8] and tumor progression [6], [9], [10], and that these events are mediated by a high affinity receptor, plexin B1, and possibly plexin B2 [11]. Most recently, we have identified Sema4D on platelets and shown that it participates in thrombus formation in vivo by reinforcing collagen-initiated platelet activation [12], [13].

Sema4D cleavage at a juxtamembrane site generates a 120-kDa soluble exodomain fragment capable of binding to and activating its receptors. After being shed from blood cells, soluble Sema4D can remain in the circulation, although its half-life is unknown. Early studies showed that Sema4D was spontaneously shed from T-cells by proteolytic enzymes and that the shed fragment can affect immune cell migration, CD40-induced B-cell proliferation, and immunoglobulin and cytokine production [14], [15], [16], [17], [18], [19]. Our previous studies on platelets [12] showed that activated platelets also shed Sema4D and that the rate of shedding is relatively slow compared to platelet aggregation, requiring nearly 30 min to reach completion. The shedding is blocked by metalloproteinase inhibitors and abolished in mouse platelets that lack the metalloproteinase ADAM17 (TACE). Mice lacking Sema4D exhibit delayed arterial occlusion after vascular injury. In separate studies, MT1-MMP (membrane-type-1 matrix metalloproteinase) has been identified as a second protease capable of cleaving the Sema4D extracellular domain. This process has been proposed to play a role in controlling tumor-induced angiogenesis [20].

In addition to its role in supporting platelet activation and B-cell development, Sema4D may participate in pathological processes. As reviewed by Ch’ng and Kumanogoh [6], several studies have described a role for Sema4D and plexin-B1 in tumor progression, including soft tissue sarcomas, B-cell chronic lymphocytic leukemia, renal cell carcinoma, breast and ovarian carcinomas, prostate adenocarcinoma, and pancreatic cancer. Sema4D or CD72 have also been reported to play a role in systemic sclerosis [21], experimental autoimmune encephalomyelitis [22], experimental crescentic glomerulonephritis [23], immune complex glomerulonephritis [24], and allergy [25]. Deletion of Sema4D in mice protects against atherosclerosis by attenuating platelet hyperactivity in the setting of hyperlipidemia [26] or by reducing intimal neovascularization [27]. Most recently, it was reported that Sema4D expression by osteoclasts suppresses bone formation [28] and that Sema4D indirectly regulates bone resorption via its effect on reproductive function [29].

Heart failure (HF) is a disease with high mortality and morbidity. The underlying disease mechanisms are complex and may involve a variety of structural and biological alterations that directly or indirectly impair cardiac function [30], [31]. The role of inflammation in the pathogenesis of heart failure is thought to involve a variety of cytokines as well as leukocytes, platelets, tissue macrophages and endothelial cells [32], [33]. Additional factors may include blood coagulation and rheological abnormalities, activation of the neuroendocrine system, and platelet abnormalities [34]. When heart failure is complicated with diabetes mellitus (DM), the prognosis is poor due to the metabolic impact of the underlying insulin resistance and hyperglycemia, which are associated with diastolic dysfunction and reduced stress tolerance [35].

Since soluble Sema4D can potentially affect both the immune and cardiovascular systems, we hypothesized that circulating Sema4D levels may be altered in HF patients. We also asked whether the concurrent presence of DM alters soluble Sema4D levels in HF patients. To test this hypothesis, we developed a new ELISA that detected soluble Sema4D in human plasma. The results showed a significant increase in plasma Sema4D levels in HF patients with the highest levels being in patients with both heart failure and diabetes.

## Materials and Methods

### Study Population

This study was approved by the ethics committees at the First and the Second Affiliated Hospitals, Soochow University, and the First People’s Hospital of Yancheng City. All participants gave written informed consent. A total of 157 HF patients from 3 hospitals in China were included. These patients were hospitalized for symptoms of heart failure such as fatigue, shortness of breath, and edema at rest or with exercise (New York Heart Association [NYHA] functional classes II, III, or IV). Some patients were previously diagnosed with heart failure and rehospitalized for acute decompensation. The mean ejection fraction in these HF patients was 48.1±17.9%, and the mean ejection fractions of patients with NYHA class II, III, and IV were 52.9±19.1, 48.4±17.1, and 44.5±17.9%, respectively. Patients with chronic obstructive lung disease, congenital heart disease, and cancer were excluded. In addition, 126 healthy subjects, who underwent routine medical check-ups at the hospitals and had no histories of cardiovascular disease, were also included. All participants were ethnic Han Chinese, the predominant population in China.

### Clinical Diagnosis

As described in our previous study [36], paper and electronic medical records were reviewed to obtain information on medical history, clinical examination, electrocardiography (ECG), echocardiography, chest X-ray, and other laboratory tests of the patients. Cardiac arrhythmia was confirmed by ECG or 24-hour Holter monitoring. Valvular heart disease was confirmed by echocardiography. As part of routine practice, all patients underwent evaluation for heart failure diagnosis and determination of disease severity by clinical history and laboratory tests including ECG. The diagnosis of heart failure and the underlying pathogenesis were determined by the cardiologists responsible for the patient care. Each patient was assigned a functional class based on the NYHA classification. Most HF patients were treated with diuretics, angiotensin-converting enzyme inhibitors, angiotensin receptor blockers, and β-blockers, according to clinical management guidelines.

### Blood Sample Collection

Peripheral venous blood was drawn into anticoagulanted tubes, as described previously [36]. Plasma samples were prepared by centrifugation at 3,000 g for 10 min, aliquoted, and either used immediately or stored at −80°C for further use within 6 months.

### ELISA Assay

Recombinant soluble human Sema4D was prepared as described [37] with the following modifications. Briefly, plasmid pSecTag2B expressing the human Sema4D extracellular domain was transfected into HEK293T cells using Lipofectamine 2000 (Life Technologies). After 4 hours, fresh medium was added and the transfected cells were cultured for 3 days. Supernatants were collected and His-tagged protein in the supernatants was purified using TALON Metal Affinity Resin (Clontech). The supernatants were incubated with the resin for 20 min at room temperature and His-tagged protein was allowed to bind the resin. After washing with a buffer (300 mmol/L sodium chloride, 50 mmol/L sodium phosphate, 10 mmol/L imidazole), the resin was packed in a column. Proteins were eluted from the column with the same buffer containing 200 mmol/L imidazole and then dialyzed against phosphate buffered saline (PBS). Purified proteins were characterized by SDS-PAGE gel, quantified using a Bradford protein assay (Bio-Rad), and then used as a standard in the following ELISA.

A sandwich ELISA was developed to detect soluble Sema4D in human plasma [38]. The purified recombinant Sema4D was used to generate monoclonal antibodies in mice. Based on an epitope analysis, one antibody was selected as a coating antibody (#4, IgG1) and another as a detecting antibody (#3, IgG1) [39]. Plates (96-wells; Corning) were coated with antibody #4 (10 µg/mL, 100 µL/well) in 50 mmol/L Na_2_CO_3_-NaHCO_3_, pH 9.6 at 4°C overnight. Wells were then blocked with 3% newborn calf serum in PBS for 2 hours. Samples and Sema4D standards were added and incubated for 1 hour at room temperature. After washing with a buffer (0.1% Tween-20 in PBS), HRP (horseradish peroxidase)-conjugated detecting antibody in 3% newborn calf serum in PBS (100 µL/well) was added and incubated at room temperature for 1 hour. One-step ultra TMB (3, 3′, 5, 5′-Tetramethylbenzidine)-substrate (Pierce) was added to the wells and incubated for 30 min in dark. Absorbance was measured at 450 nm by a plate-reader (Spectra Max M5, Molecular Devices).

The stability of soluble Sema4D in plasma samples was tested as described previously [40]. Pooled human plasma from 20 individuals with EDTA as an anticoagulant was measured for soluble Sema4D after being stored at 4°C for different time periods (0, 8, 12, 24, 48, and 72 h) or frozen and thawed for several cycles. All samples tested had similar Sema4D levels as that of pooled fresh sample (data not shown). Therefore, soluble Sema4D in plasma was stable up to 72 h in 4°C storage condition or 5 cycles of freezing and thawing. As a practical matter for the studies included in this manuscript, plasma Sema4D levels were either determined immediately or after storage at −80°C for no more than 6 months.

### Platelet Aggregation and Western Blot

Venous blood from healthy donors was anti-coagulated 1∶5 with ACD (65 mmol/L Na_3_Citrate, 70 mmol/L citric acid, 100 mmol/L dextrose, pH 4.4) and centrifuged at 900 rpm for 20 min to obtain platelet-rich plasma (PRP) [13]. Gel-filtered platelets were prepared as described previously [41]. Platelets (2×10^8^/mL) were pre-incubated with or without 17β-estradiol for 3 min and stimulated with collagen or ADP at 37°C under constant stirring at 1,000 rpm. Platelet aggregation was monitored in a lumi-aggregation meter (Chrono-Log) for 8 min and the reaction was stopped by adding cold RIPA (Radio Immunoprecipitation Assay) buffer with protease inhibitors (Roche). Platelet lysates were boiled for 10 min and proteins were separated on 10% SDS-PAGE gels, transferred to a polyvinylidenefluoride (PVDF) membrane, and immunoblotted with a purified mouse anti-Sema4D antibody (BD Transduction Laboratories) followed by a goat anti-mouse IRDye 680 or 800 antibody (LI-COR Biosciences, Lincoln, NE).

### Flow Cytometry

Peripheral blood mononuclear cells (PBMCs) were isolated from anti-coagulated blood by HISTOPAQUE-1077 (Sigma-Aldrich) gradient centrifugation. PBMCs were double-stained using an anti-Sema4D-PE antibody (A8, Abcam, Cambridge, UK) together with either an anti-CD3-FITC, anti-CD4-FITC, anti-CD8-FITC, or anti-CD19-FITC monoclonal antibody (BD Biosciences, Erembodegem, Belgium). Jurkat cells were cultured in RPMI-1640 medium with 10% of FBS (fetal bovine serum) (Hyclone) and stained using an anti-CD100-PE antibody. Background fluorescence was determined using an isotypic antibody control (BD Biosciences). Cells were analyzed using a flow cytometer (BD Biosciences). Sema4D expression on platelet surface was measured in whole blood double stained with anti-Sema4D-PE and anti-CD41-FITC antibodies.

### Statistical Analysis

The analysis was performed using SPSS (version18) and GraphPad Prism5 software. Data are presented as mean ± SD. Comparisons of plasma Sema4D levels in healthy controls and patient groups were performed using ANOVA followed by a Tukey post test. All probabilities were 2-tailed, and *p* values of <0.05 were considered statistically significant.

## Results

### ELISA Assay for Soluble Sema4D

**Figure 1:**
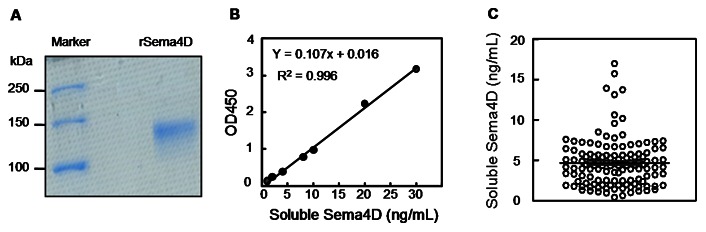
Establishment of a sandwish ELISA to measure soluble Sema4D in human plasma. A. pSecTag2B plasmid containing His-tagged Sema4D coding sequence for amino acids 1–734 was expressed in HEK293T cells. His-tagged Sema4D in the conditioned medium was purified by affinity chromatography. The recombinant Sema4D (rSema4D) migrated as an approximately 120-kDa protein in SDS-PAGE gel. B. An ELISA was developed using coating antibody #4, detecting antibody #3 conjugated with HRP, and rSema4D as a standard. The standard curve was generated under optimized conditions (capture antibody, 10 µg/mL; detection antibody, 200 ng/mL). C. Healthy donors (n = 126), who underwent routine medical check-ups and had no medical history of cardiovascular diseases, were examined for their plasma soluble Sema4D levels as described in Materials and Methods.

A human recombinant Sema4D (rSema4D) extracellular fragment containing amino acids 1–734 was expressed and purified. On SDS-PAGE gels under reducing conditions, the purified protein appeared as a single band of approximately 120 kDa (Figure 1A). To measure Sema4D in human plasma, an ELISA assay was established using two monoclonal antibodies recognizing different epitopes in the Sema4D extracellular region [39]. The assay had a detection limit of 0.53±0.03 ng Sema4D/mL of human plasma, as determined by the average concentration of the background 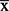
+2SD, and a linear range of 0.56–30 ng/mL of plasma Sema4D (Figure 1B).

### Soluble Sema4D in Human Plasma

**Figure 2:**
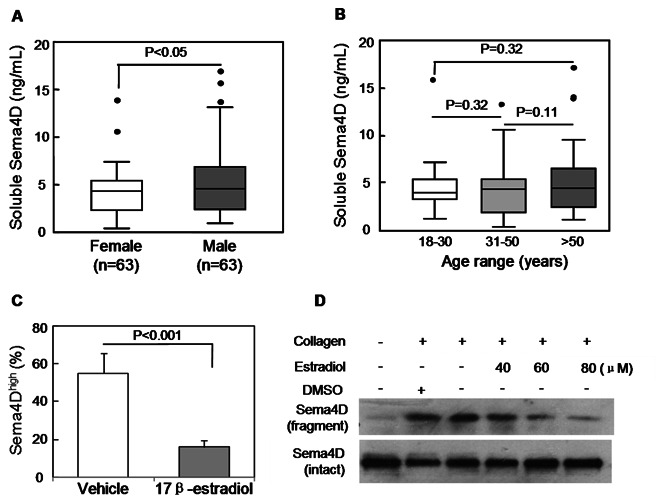
Plasma Sema4D levels in different gender and age groups of healthy donors, and the effects of 17β-estradiol on Sema4D expression in Jurkat cells and Sema4D shedding in human platelets. A. Plasma Sema4D levels in healthy males and females. B. Plasma Sema4D levels in healthy donors of different age groups. Each box represents the median and interquartile range values. The outliers that are >1.5 the interquartile range are indicated by filled circles. The vertical bars indicate the non-outlier minimum and maximum. C. The percentages of Sema4D^high^ Jurkat cells with or without 10 µM 17β-estradiol treatments were measured by flow cytometry. P values were obtained using ANOVA followed by a Tukey post test. D. Gel-filtered platelets were prepared from healthy donors and pre-incubated with 0.5% DMSO (vehicle control) or 17β-estradiol (40 µM, 60 µM and 80 µM) for 3 min. Collagen (2 µg/mL) was used to induce platelet aggregation for 8 min. Platelet samples were analyzed by Western blot using a Sema4D C-terminal antibody to visualize the Sema4D cleavage product. Data shown are representative of at least 3 independent experiments.

In 126 healthy individuals, plasma Sema4D levels were 4.67±2.99 ng/mL (Figure 1C). There was a small but statistically significant gender difference: levels of plasma Sema4D in men (5.15±3.30 ng/mL, n = 63) were higher than that in women (4.19±2.39 ng/mL, n = 63, *P*<0.05) (Figure 2A). There were no significant age-related differences (Figure 2B).

### Effect of Estradiol on Sema4D Expression and Shedding

To test the hypothesis that estrogen may influence plasma Sema4D levels, we studied the effect of 17β-estradiol on Sema4D expression in T cell-derived Jurkat cells. Flow cytometry showed that Sema4D expression in Jurkat cells was decreased in the presence of 17β-estradiol (*P*<0.001) (Figure 2C). We also examined Sema4D shedding by human platelets [12] and found that pre-incubation of platelets with 17β-estradiol markedly inhibited Sema4D shedding induced by collagen (Figure 2D). These results suggest that estrogen and its derivatives may inhibit both Sema4D expression and shedding.

### Increased Plasma Sema4D Levels in HF Patients

**Figure 3:**
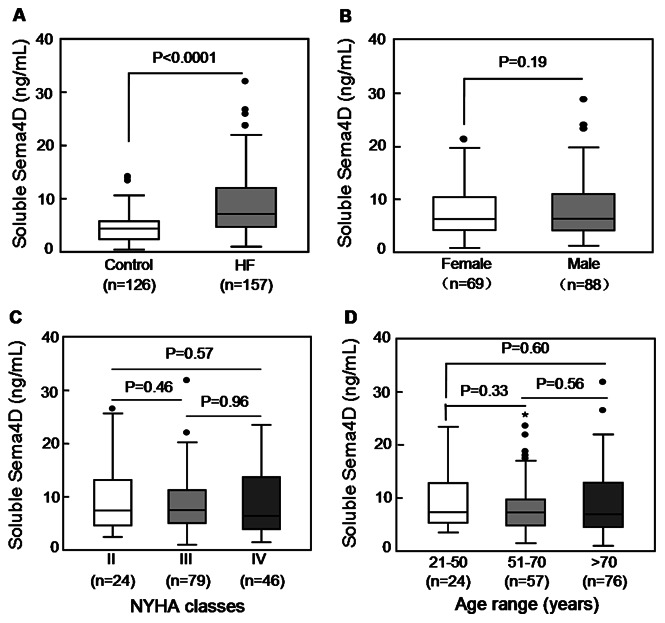
Plasma soluble Sema4D levels in HF patients. A total of 157 HF patients were studied. A. Comparison of plasma Sema4D levels between HF patients and healthy donors. B. Comparison of plasma Sema4D levels between male and female HF patients. C. Comparison of plasma Sema4D levels in HF patients of NYHA functional classes II, III, and IV. D. Comparison of plasma Sema4D levels in HF patients of different age groups. Each box represents the median and interquartile range values. The outliers that are >1.5 and >3 times the interquartile range are indicated by filled circles and stars, respectively. The vertical bars indicate the non-outlier minimum and maximum. *P* values were obtained using ANOVA followed by a Tukey post test.

Next, we measured plasma Sema4D levels in HF patients. The main characteristics of the 157 HF patients in this study are summarized in Table 1. The levels of plasma Sema4D in HF patients (8.94±5.89 ng/mL) were significantly higher than those of healthy controls (4.67±2.99 ng/mL) (*P*<0.0001) (Figure 3A). In contrast to the healthy controls, there was no significant gender difference in plasma Sema4D levels in HF patients (9.26±6.35 ng/mL in males n = 88 *vs*. 8.54±5.26 ng/mL in females, n = 69, *P*>0.05) (Figure 3B). To test whether the increased Sema4D levels correlate with the disease severity, we grouped HF patients into three NYHA classes (class II, n = 24; class III, n = 79; class IV, n = 46). Plasma Sema4D levels were 9.83±7.07, 8.83±5.39, and 8.89±6.31 ng/mL, respectively. None of these differences are statistically significant (Figure 3C; *P* values >0.5). There was also no correlation between ejection fraction and soluble Sema4D levels (analysis not shown). As in the healthy individuals, plasma Sema4D levels did not differ in different age groups (Figure 3D).

**Table 1:** Characteristics of healthy donors and HF patients.

Characteristics	Control (n = 126)	HF (n = 157)
Age, mean (SD)	44.7 (11.9)	66.2 (14.9)
Sex, n (%)		
male	63 (50)	88 (56.1)
female	63 (50)	69 (43.9)
Medical history, n (%)		
Hypertension	0 (0)	94 (59.9)
Diabetes mellitus	0 (0)	40 (25.5)
Valvular heart disease	0 (0)	17 (10.8)
Cardiomyopathy	0 (0)	20 (12.7)
Coronary artery disease	0 (0)	24 (15.3)
Cardiac arrhythmia	0 (0)	18 (11.5)
Others	0 (0)	23 (14.6)

### Increased Plasma Sema4D Levels in HF Patients with Diabetes

**Figure 4:**
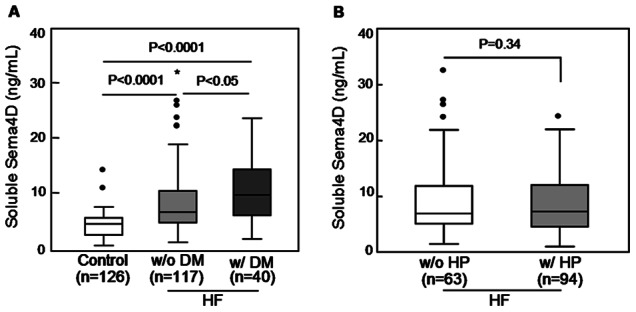
Plasma Sema4D levels in HF patients with diabetes or hypertension. A. Plasma Sema4D levels in HF patients with or without diabetes mellitus (DM). B. Plasma Sema4D levels in HF patients with or without hypertension (HP) history. Each box represents the median and interquartile range values. The outliers that are >1.5 and >3 times the interquartile range are indicated by filled circles and stars, respectively. The vertical bars indicate the non-outlier minimum and maximum. *P* values were obtained using ANOVA followed by a Tukey post test.

DM and hypertension are known risk factors for heart failure. We analyzed whether these risk factors correlated with the increased plasma Sema4D levels. As shown in Figure 4A, HF patients with diabetes (10.45±5.76 ng/mL, n = 40) or without diabetes (8.42±5.87 ng/mL, n = 117) had higher plasma Sema4D levels than healthy controls (4.67±2.99 ng/mL, n = 126) (both P values <0.01). Levels were highest in patients with diabetes. In contrast, levels were similar in HF patients with or without hypertension (Figure 4B).

### Increased Sema4D-positive T Cells but not B Cells or Platelets in HF Patients

**Figure 5:**
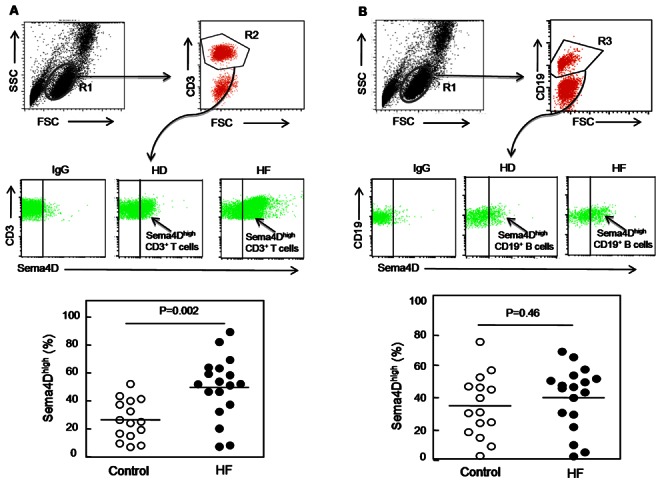
Sema4D expression on lymphocytes in HF patients and healthy donors. Fresh PBMCs from HF patients and healthy donors were double-stained and analyzed by FACS. The region corresponding to lymphocytes (R1) was selected using an FSC-H/SSC-H density plot. Using an FITC-density plot applied to the R1 region, the regions R2 and R3 corresponding to CD3^+^ and CD19^+^ cells, respectively, were defined (upper panels of 5A and 5B). In the PE density plot (middle panels of 5A and 5B), the right region delimits CD3^+^ and CD19^+^ cells with high expression of Sema4D, respectively. PE-conjugated IgG was used as an isotypic control. The percentage of Sema4D^high^ CD3^+^ cells and Sema4D^high^ CD19^+^ cells in healthy donors (HD) and HF patients (HF) were analyzed (lower panels of 5A and 5B). *P* values were obtained using ANOVA followed by a Tukey post test.

**Figure 6:**
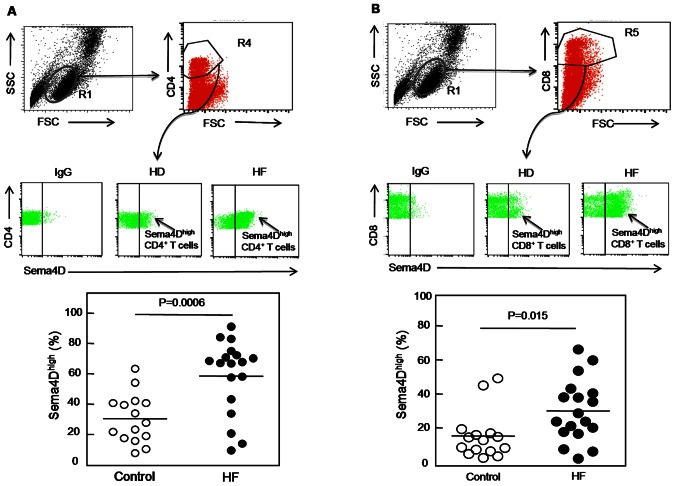
Sema4D expression on T cell subpopulations in HF patients and healthy donors. Fresh PBMCs from HF patients and healthy donors were double-stained and analyzed by FACS. The region corresponding to lymphocytes (R1) was selected using an FSC-H/SSC-H density plot. Using a FITC-density plot applied to the R1 region, the regions R4 and R5 corresponding to CD4^+^ and CD8^+^ cells, respectively, were defined (upper panels of 6A and 6B). In the PE density plot (middle panels of 6A and 6B), the right region delimits CD4^+^ and CD8^+^ cells with high expression of Sema4D, respectively. PE-conjugated IgG was used as an isotypic control. The percentage of Sema4D^high^ CD4^+^ cells and Sema4D^high^ CD8^+^ cells in healthy donors (HD) and HF patients (HF) were analyzed (lower panels of 6A and 6B). *P* values were obtained using ANOVA followed by a Tukey post test.

Lymphocytes and platelets [12] are the primary cells expressing Sema4D in the hematopoietic system. We hypothesized that increased plasma Sema4D levels in HF patients may be due to increased Sema4D expression and/or shedding in circulating lymphocytes or platelets. Interestingly, although overall lymphocyte numbers were unaffected, the percentage of Sema4D^high^ CD3^+^ T-cells was significantly higher in HF patients than healthy donors (*P* = 0.002) (Figure 5A). Such a difference was not observed in CD19^+^B cells (*P* = 0.46) in these two groups (Figure 5B). These results indicate that Sema4D-positive T lymphocytes were increased in HF patients, which may contribute to the increased plasma Sema4D levels in these patients. We also examined T cell subpopulations and found that Sema4D expression in both Sema4D^high^ CD4^+^ (*P* = 0.0006) (Figure 6A) and Sema4D^high^ CD8^+^ (*P* = 0.015) (Figure 6B) T cells was significantly higher in HF patients than healthy controls.

**Figure 7:**
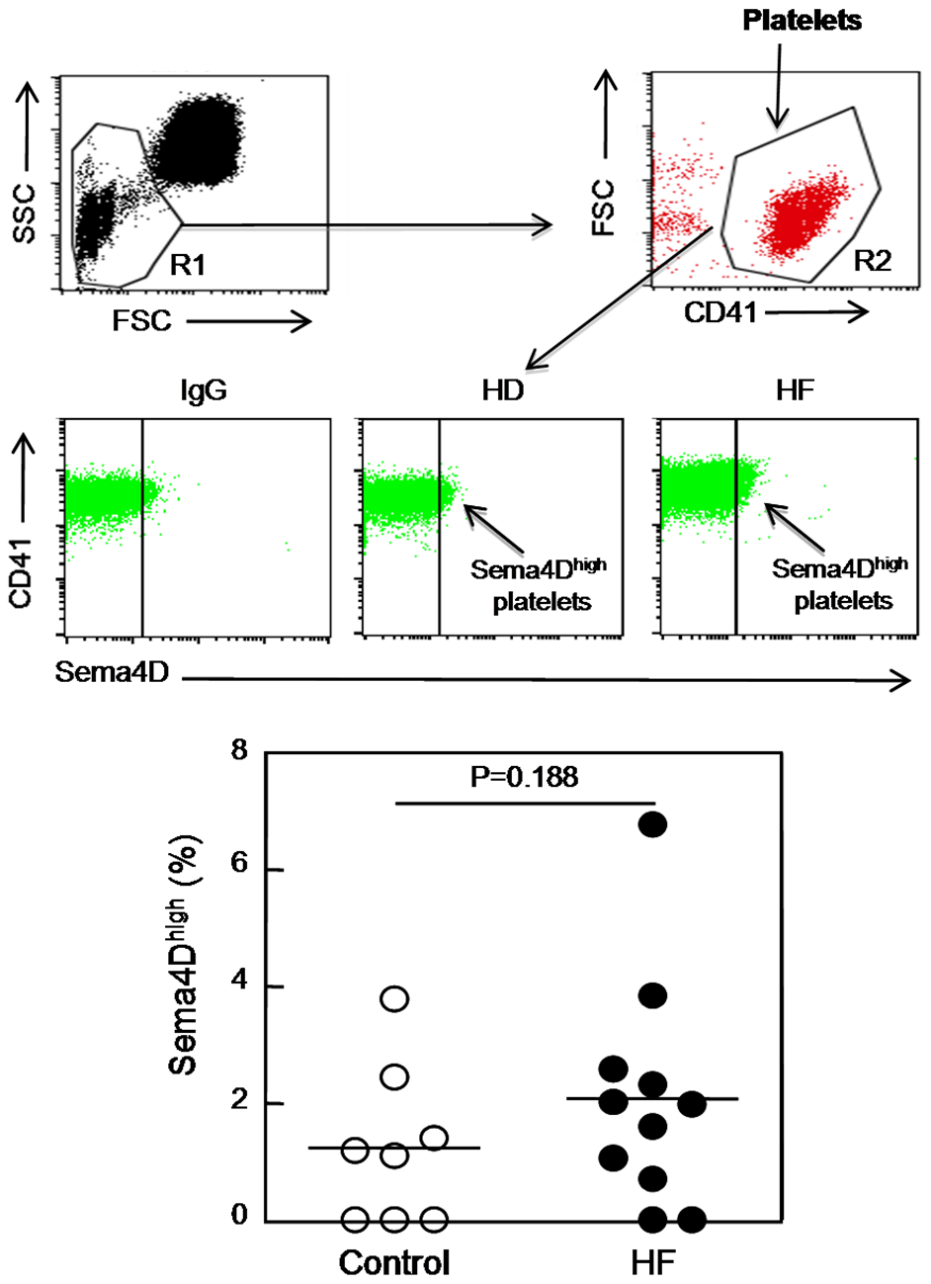
Sema4D expression on platelet surface in HF patients and healthy donors. Whole blood diluted 1:20 in Tyrode buffer was double-stained and analyzed by FACS. Using a FITC-density plot applied to the R1 region, the region R2 corresponding to platelets was defined (upper panels). In the PE density plot (middle panels), the right region delimits platelets with high expression of Sema4D in healthy individuals or HF patients. PE-conjugated IgG was used as an isotypic control. The percentage of Sema4D^high^ platelets in HF patients and healthy donors (HD) was presented in dotted diagrams (lower panel). *P* values were obtained using ANOVA followed by a Tukey post test.

To evaluate whether platelet Sema4D contributes to the increased levels of soluble Sema4D in HF patients, we isolated platelets from HF patients and health donors, and measured surface expression of Sema4D. Platelets were double-stained with anti-CD41 and anti-Sema4D antibodies. HF patients had slightly higher levels of soluble Sema4D^high^ platelets than healthy individuals (Figure 7). The difference, however, was not statistically significant (*P* = 0.188).

## Discussion

Lymphocyte and platelet activation increases Sema4D expression and subsequent proteolytic cleavage to generate soluble forms of Sema4D [12], [42]. Plasma Sema4D levels may vary under pathological conditions. Several studies [14], [17], [21] have used ELISA to measure Sema4D in human and mouse serum or cell culture media. However, measurements made in serum samples are potentially confounded by the release of Sema4D from platelets ex vivo during preparation of the serum from whole blood samples, making it difficult to use the data to interpret plasma Sema4D levels. Here we focused on plasma Sema4D levels, which may better reflect circulating soluble Sema4D levels in blood. We developed an ELISA using two antibodies against different epitopes in the Sema4D extracellular domain and detected Sema4D in human plasma.

Unexpectedly, we found that the levels were lower in healthy females than males, suggesting that sex hormones may affect Sema4D expression and/or shedding. Indeed, we found that 17β-estradiol, a potent estrogen derivative, inhibited Sema4D expression in T cell-derived Jurkat cells and collagen-induced Sema4D shedding in human platelets. Possibly, estrogen and its derivatives may inhibit Sema4D expression and shedding *in vivo*, resulting in the small gender difference observed in healthy subjects.

An important finding of this study is that plasma Sema4D levels were significantly higher in HF patients than healthy controls. Unlike in healthy controls, we did not observe a significant gender difference in plasma Sema4D levels in HF patients. Since plasma Sema4D levels were significantly elevated in both male and female HF patients, the increase may have obscured difference that occurs in healthy adults. Alternatively, the inhibitory effect of female hormones on Sema4D expression and/or shedding may be counter-balanced by other neural hormonal changes that may occur in HF patients.

To understand the mechanism underlying the elevated plasma Sema4D levels in HF patients, we analyzed platelets and T cells, two major cellular sources of Sema4D in blood. By flow cytometry, we did not find significant differences in platelet Sema4D levels between HF patients and healthy controls, suggesting that other blood cells, possibly T cells, are the primary source of increased plasma Sema4D levels in HF patients. Indeed, we found that HF patients had a significantly increased T cell subpopulation that was Sema4D positive, which included Sema4D^high^ CD3^+^, Sema4D^high^ CD4^+^, and Sema4D^high^ CD8^+^ cells. In contrast, we did not detect major differences in CD19^+^ B cell population between HF patients and healthy controls. These results indicate that a subpopulation of Sema4D-positive T cells was increased in HF patients and may contribute to the high levels of Sema4D in patients’ plasma. Further investigation on the relation of Sema4D^high^ T cells with markers of inflammation and cytokine levels in heart failure would help understand the mechanism of this cardiovascular disorder.

Sema4D is an integral membrane protein. Metalloproteinase ADAM17 and MT1-MMP have been identified to shed Sema4D from the surface of platelets and cancer cells [12], [20]. ADAM17 is known to cleave its substrates TNFα receptor and L-selectin in T cells [43]. Similarly, MT1-MMP is also expressed in T cells [44]. In platelets, ADAM17 is the primary enzyme responsible for Sema4D shedding, as indicated by the lack of Sema4D shedding in the absence of ADAM17 [12]. Studies have shown that ADAM17 and MT-MMP1 expression is up-regulated in cardiomyocytes and PBMCs in patients with heart disease [45], [46], [47]. It is possible that increased activities of ADAM17 and/or MT1-MMP promoted Sema4D shedding from T cells in HF patients, thereby contributing to the elevated levels of plasma Sema4D.

Another notable finding of this study is that plasma Sema4D levels in HF patients with diabetes were even higher than those in HF patients without diabetes. Diabetes is a chronic progressive metabolic disease. Two Sema4D receptors, plexins B1 and B2, have been identified in pancreatic epithelial ducts of mouse embryos. Their expression appeared to co-localize with both insulin-producing β cells and glucagon-producing α cells [48]. Similar findings were reported in human pancreas, where Sema4D-positive infiltrating lymphocytes interacted with plexin B1-positive tumor cells in pancreatic ducts [49]. It is possible that in HF patients an increased Sema4D-positive T cell subpopulation may target pancreatic cells and reduce insulin production. In addition, Sema4D may exacerbate vascular complications in DM patients by interacting with endothelial cells to promote vascular inflammation, endothelial dysfunction, microthrombus formation and atherosclerosis [50].

In summary, we report here that soluble Sema4D is present in the plasma of healthy individuals and that the level was increased in HF patients, especially in those with concurrent diabetes. The increase in plasma Sema4D levels was associated with an increase in Sema4D-positive T-cells in HF patients. These results suggest a possible mechanism, by which plasma soluble Sema4D and/or Sema4D-positive T-cells may contribute to the development and/or progression of heart failure and diabetes in patients. Our study has its limitations because of its retrospective nature and a relatively small set of patient samples. Conversely, HF and diabetes may result in high levels of plasma Sema4D, which in turn may exacerbate HF and diabetes. Our findings should encourage future studies in animal models and with larger cohorts of patients to understand the role of Sema4D in heart failure and diabetes.
