# Correction: Increased Levels of Plasma Soluble Sema4D in Patients with Heart Failure

**DOI:** 10.1371/journal.pone.0214894

**Published:** 2019-03-28

**Authors:** Qiongyu Lu, Ningzheng Dong, Qi Wang, Wenxiu Yi, Yuxin Wang, Shengjie Zhang, Haibo Gu, Xin Zhao, Xiaorong Tang, Boquan Jin, Qingyu Wu, Lawrence F. Brass, Li Zhu

There are errors in Fig 5 and Fig 6. Please see the correct Fig 5 and Fig 6 here.

**Fig 5:**
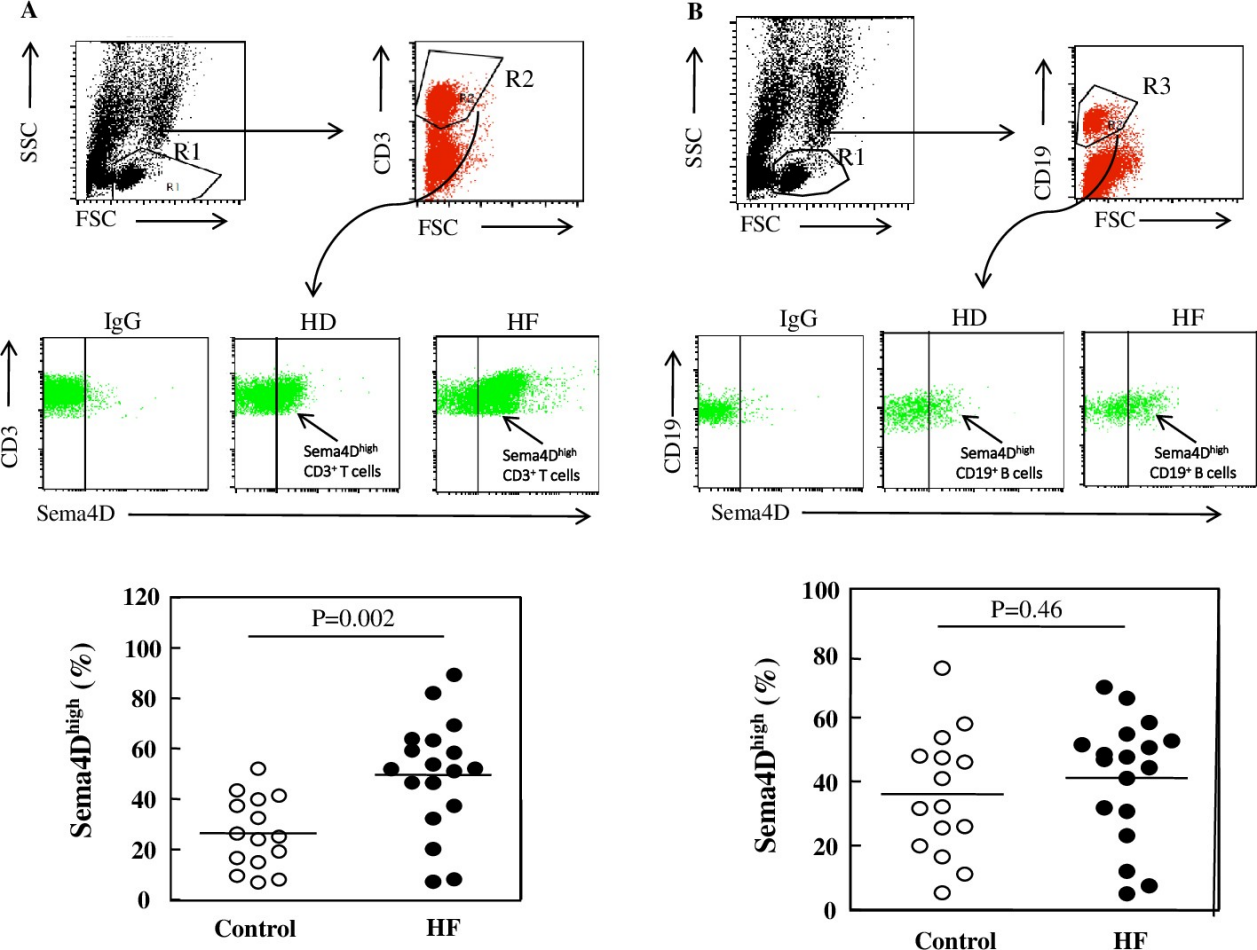
Sema4D expression on lymphocytes in HF patients and healthy donors. Fresh PBMCs from HF patients and healthy donors were double-stained and analyzed by FACS. The region corresponding to lymphocytes (R1) was selected using an FSC-H/SSC-H density plot. Using an FITC-density plot applied to the R1 region, the regions R2 and R3 corresponding to CD3^+^ and CD19^+^ cells, respectively, were defined (upper panels of 5A and 5B). In the PE density plot (middle panels of 5A and 5B), the right region delimits CD3^+^ and CD19^+^ cells with high expression of Sema4D, respectively. PE-conjugated IgG was used as an isotypic control. The percentage of Sema4D^high^ CD3^+^ cells and Sema4D^high^ CD19^+^ cells in healthy donors (HD) and HF patients (HF) were analyzed (lower panels of 5A and 5B). *P* values were obtained using ANOVA followed by a Tukey post test.

**Fig 6:**
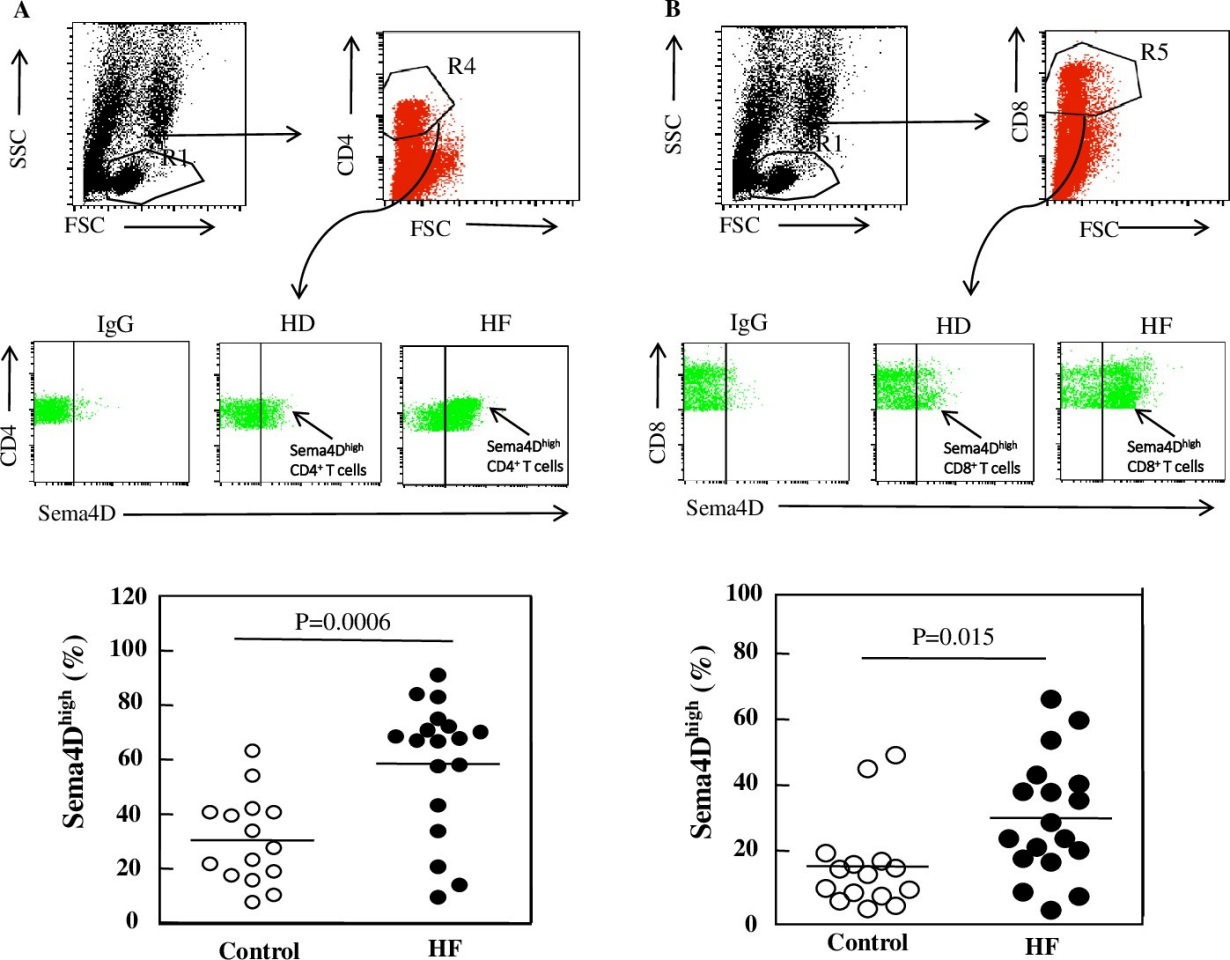
Sema4D expression on T cell subpopulations in HF patients and healthy donors. Fresh PBMCs from HF patients and healthy donors were double-stained and analyzed by FACS. The region corresponding to lymphocytes (R1) was selected using an FSC-H/SSC-H density plot. Using a FITC-density plot applied to the R1 region, the regions R4 and R5 corresponding to CD4^+^ and CD8^+^ cells, respectively, were defined (upper panels of 6A and 6B). In the PE density plot (middle panels of 6A and 6B), the right region delimits CD4^+^ and CD8^+^ cells with high expression of Sema4D, respectively. PE-conjugated IgG was used as an isotypic control. The percentage of Sema4D^high^ CD4^+^ cells and Sema4D^high^ CD8^+^ cells in healthy donors (HD) and HF patients (HF) were analyzed (lower panels of 6A and 6B). *P* values were obtained using ANOVA followed by a Tukey post test.

## References

[1] Lu Q, Dong N, Wang Q, Yi W, Wang Y, Zhang S, et al (2013) Increased Levels of Plasma Soluble Sema4D in Patients with Heart Failure. PLoS ONE 8(5): e64265 10.1371/journal.pone.0064265 23741311 PMC3669357

